# Phenotypic plasticity in female mate choice behavior is mediated by an interaction of direct and indirect genetic effects in *Drosophila melanogaster*

**DOI:** 10.1002/ece3.2954

**Published:** 2017-04-09

**Authors:** David C. S. Filice, Tristan A. F. Long

**Affiliations:** ^1^ Department of BiologyWilfrid Laurier UniversityWaterlooONCanada

**Keywords:** indirect genetic effects, mate choice, phenotypic plasticity, population genetics, sexual conflict, sexual selection

## Abstract

Female mate choice is a complex decision‐making process that involves many context‐dependent factors. In *Drosophila melanogaster*, a model species for the study of sexual selection, indirect genetic effects (IGEs) of general social interactions can influence female mate choice behaviors, but the potential impacts of IGEs associated with mating experiences are poorly understood. Here, we examined whether the IGEs associated with a previous mating experience had an effect on subsequent female mate choice behaviors and quantified the degree of additive genetic variation associated with this effect. Females from 21 different genetic backgrounds were housed with males from one of two distinct genetic backgrounds for either a short (3 hr) or long (48 hr) exposure period and their subsequent mate choice behaviors were scored. We found that the genetic identity of a previous mate significantly influenced a female's subsequent interest in males and preference of males. Additionally, a hemiclonal analysis revealed significant additive genetic variation associated with experience‐dependent mate choice behaviors, indicating a genotype‐by‐environment interaction for both of these parameters. We discuss the significance of these results with regard to the evolution of plasticity in female mate choice behaviors and the maintenance of variation in harmful male traits.

## 1 Introduction

Female mate choice is a powerful agent of selection that has led to the evolution of exaggerated male display traits and dimorphisms between the sexes in many taxa (Andersson, 1994; Darwin, 1871). Within species, females often exhibit considerable individual variation in their choice of mates, the extent of which influences the strength and direction of evolution via sexual selection, and consequently the potential for population change, divergence, and speciation (Hebets & Sullivan‐Beckers, 2010; Jennions & Petrie, 1997; Verzijden et al., 2012). Due to these important evolutionary consequences, identifying the specific environmental and/or genetic sources of individual variation in female mate choice behaviors is of great interest to biologists. One potentially important source of this variation is behavioral plasticity mediated by individual social experience (Bailey & Moore, 2012; Rodríguez, Rebar, & Fowler‐Finn, 2013; Travers, Simmons, & Garcia‐Gonzalez, 2016). Behavioral plasticity refers to a change in an individual's behavior (such as mate choice) in response to local environmental conditions (Mery & Burns, 2010). Social experience may act as one of the conditions that results in the modification of an individual's behavior. Despite its potential importance as a source of individual variation in female mating behaviors, few studies have explored the causes and consequences of behavioral plasticity in the context of social and mating experience (Rodríguez, Rebar, et al., 2013). Additionally, little is known about the degree to which this type of socially mediated plasticity is influenced by individual genetic variation (i.e., a genotype‐by‐environment interaction) (Ingleby, Hunt, & Hosken, 2010). Here, we examine how the expression of mate choice behaviors in females from different genetic backgrounds may vary in response to different mating histories.

In some species, plasticity in female mate choice behaviors depends on an organism's specific environmental history (Hunt, Brooks, & Jennions, 2005; Hebets, Wesson, & Shamble, 2008; Dukas, 2005; Bailey & Zuk, 2009; Rebar, Zuk, & Bailey, 2011; Travers et al., 2016). As mate choice is often a costly behavior (Kokko et al., 2003), it is possible that the expression of mate choice is subject to trade‐offs under different contexts, in a similar manner as other life‐history traits such as body size (Hunt et al., 2005). Qvarnström (2001) argued that in some circumstances, plasticity in mate choice behaviors may be actually favored by selection over static behaviors, as—in some situations—the expenditure of resources and the risk of injury or predation associated with choosing between potential mates may come at a greater cost to the individual than the benefits that arising from being choosy. If a female expresses plasticity in her mate choice behaviors, she could potentially maximize her potential reproductive success in a wide range of environmental contexts (Qvarnström, 2001). One such context may be an individuals’ social environment and/or their mating history. In Pacific field crickets (*Teleogryllus oceanicus*), females modify the expression of both their pre‐ and post‐copulatory choice mechanisms depending on their previous mating experience (Rebar et al., 2011). Females that had previously mated to an “attractive” male 24 hr earlier mounted new males more slowly compared to those females that had previously mated with an “unattractive” male, suggesting that individual females may use the information about the attractiveness of their previous mates when evaluating the potential suitability of their subsequent potential mates (Rebar et al., 2011). This type of plasticity may help a female ensure that she is choosing the most attractive mate available in her social environment (Rebar et al., 2011).

In addition to social experiences, an individual's behavioral expression may be shaped by the specific genotypes of interacting conspecifics via indirect genetic effects (IGEs *sensu* Rodríguez, Rebar, et al., 2013), or through interactions between their own genotype and their environment (GxEs). IGEs arise when the genes expressed by an individual influence the phenotype of a conspecific, typically via social interactions (Wolf, Brodie, Cheverud, Moore, & Wade, 1998). Previous studies have shown that IGEs are involved in female mate choice behavioral variation in the field cricket, *T. oceanicus* (Bailey & Zuk, 2012), and in the tree hopper, *Enchenopa binotata* (Rebar & Rodríguez, 2013). In the latter case, individuals housed with different families exhibited variation in their mate preferences, suggesting that not only does social experience influence the expression of mate choice behavior, but so does the genotype of the interacting individuals. In contrast, GxEs arise when the phenotypic expression of a genotype varies depending on the environments in which they are expressed (Lynch & Walsh, 1998). Interactions between genetic and environmental factors have been shown to affect the expression of sexual traits (e.g., courtship behavior (Etges et al., 2007) and sperm length (Morrow, Leijon, & Meerupati, 2008)), but few studies have demonstrated that female choice can similarly be affected (Ingleby et al., 2010). Identifying these interactions is important, because in order for plasticity in mate choice to evolve, there must be genetic variation in the way individuals behaviorally respond to environmental factors (Ingleby et al., 2010). To date, GxE effects in mate choice have only been identified in the lesser waxmoth, *Achroia grisella* (Rodríguez & Greenfield, 2003), the fruit fly, *Drosophila melanogaster* (Narraway et al., 2010), and the treehopper, *E. binotata* (Rodríguez, Hallett, Kilmer, & Fowler‐Finn, 2013). In the case of fruit flies, Narraway et al. (2010) identified variation in female choice across isogenic females that were either exposed to a their regular rearing temperature (25°C) or experienced a cold (4°C cold shock for 15 min day^−1^, for 10 days) environment. Although these studies are a good start, researchers have only tested for GxEs in mate choice by manipulating abiotic environmental factors. As such, we set out to directly test whether the IGEs associated with social experiences can interact with genotype to produce individual variation in female mate choice behaviors.

When considering the sources of plasticity in female mate choice behaviors, previous research has primarily focused on IGEs associated with general social experiences (Bailey & Zuk, 2012; Rebar & Rodríguez, 2013). However, the role of IGEs associated with individual physical mating experience has not received any specific study. In many species, males may physically harm their mates as a pleiotropic side effect of traits that have been selected to increase individual male success, a process known as sexual conflict (Morrow, Arnqvist, & Pitnick, 2003). This harm can be costly to a female's lifetime fitness as it may involve physical genitalia damage (Kamimura, 2007) and may reduce both her longevity and lifetime fecundity (Filice & Long, 2016; Lew & Rice, 2005; Partridge & Fowler, 1990). Sexual conflict is predicted to arise whenever males and females of the same species have different (and incompatible) strategies for maximizing their lifetime reproductive success (Parker, 1979; Chapman, Arnqvist, Bangham, & Rowe, 2003; Arnqvist & Rowe, 2005). In the fruit fly, *D. melanogaster*, a model species for the study of sexual selection and conflict, mating can elicit numerous changes in a female's physiology and behavior (Bonduriansky & Day, 2013; Chapman & Davies, 2004; Wong & Wolfner, 2006). Many of these changes are induced by the toxicity of accessory gland proteins (Acps) that are transferred in a male's ejaculate during copulation (Chapman, Liddle, Kalb, Wolfner, & Partridge, 1995; Rice, 1996). Acps can influence female phenotypes by increasing short‐term egg production (Soller et al., 1999), decreasing future mating receptivity (Chen et al., 1988; Chapman et al., 2003), shortening lifespan (Chapman et al., 1995), and increasing feeding behavior (Carvalho et al., 2006). Substantial evidence indicates individual variation in the degree of these male‐induced effects has an additive genetic basis and that IGEs associated with mating can influence important components of a female's fitness such as fecundity and longevity (Filice & Long, 2016; Fiumera, Dumont, & Clark, 2006; Friberg, 2005; Lew & Rice, 2005).

In this study, we attempt to synthesize the effects of sexual conflict with our understanding of IGEs on plasticity in mate choice behaviors. If males vary in the magnitude of their physiological and behavioral effects on females via mating, we predicted that female mate choice behaviors will differ depending on the phenotypes expressed by her previous mate. To test this prediction, we examined whether female mate choice behaviors varied depending on the duration of her exposure to males and to the genotype of these mates (i.e., IGEs). Furthermore, as *D. melanogaster* females exhibit genetic variation in how they rank male “attractiveness” and in how much they discriminate against certain males (Ratterman, Rosenthal, Carney, & Jones, 2014; *but see* Tennant, Sonser, & Long, 2014), we also examined whether there was genetic variation associated with female behavioral response to mating experience. If there is a genetic basis for individual variation in female mate choice behaviors, then we predicted we would observe heterogeneity in the magnitude of the experience‐mediated behavioral plasticity (i.e., a GxE interaction). By studying the potential role that the interaction between female genotype and mating experience contributes to individual variation in female male choice, this study helps advance our understanding of the complex nature of evolutionary change via sexual selection.

## 2 Methods

### 2.1 Fly stock and hemiclone generation

The flies used in our experiments were derived from the large outbred wild‐type *Ives* (hereafter “IV”) population. This population originated from a sample of 200 females and 200 males collected in South Amherst, MA, USA, 1975. Since 1980, this population has been cultured at a large census size (>1,000 adults/generation) on nonoverlapping generations and standardized protocols (Rose et al. 1984; Long, Montgomerie, & Chippindale, 2006; Martin & Long, 2015; Filice & Long, 2016). Flies are cultured in vials containing 10 ml of a banana‐agar‐killed yeast medium, and raised in an incubator that maintains a consistent 25°C, 60%, humidity environment on a 12‐hr:12‐hr light:dark diurnal cycle. At the start of each generation, flies are collected from their “natal” vials as adults, mixed *en masse*, and transferred into equal groups into “oviposition” vials containing fresh media. Flies are left in these vials for ~2–3 hr to allow oviposition. After this period, the adult flies are removed and the eggs that were laid are trimmed (by hand) to a density of 100 eggs/vial. These oviposition vials become the natal vials for the next generation of flies.

From the IV population, we established 26 male clone lines using cytogenetic cloning techniques (Chippindale, Gibson, & Rice, 2001; Abbott & Morrow, 2011; Tennant et al., 2014), which were subsequently expressed in either a male or female “hemiclonal” background. Clone lines were initially created and subsequently maintained by mating males chosen from the IV population to females from a “clone‐generator” (CG) population, who possess a random Y chromosome, a conjoined “double‐X” chromosome [C(1)DX, *y, f*], and are homozygous for translocated autosomes [T(2;3) *rdgC st in ri p* ^P^ *bw* ^D^]. The resulting males in each clone line all possess one full haplotype originating from the base IV population maintained in an unrecombined state and with the translocated autosomes inherited from their CG mother. To express the haploid genome in a male hemiclonal state, clone males are crossed with virgin females from the “DX‐IV” population (which possess the “double‐X” chromosome and autosomes originating from the IV population). To express the haploid genome in a female hemiclonal state, clone males are crossed to virgin females from the base IV population. Ultimately, all target hemiclones resulting from one of these crosses posses one haplotype identical to all other individuals in the line, and one randomly inherited haplotype.

Prior to this study, we quantified the magnitude of the effect of male exposure on female fecundity in the 26 hemiclone lines by measuring egg production (a meaningful metric of fitness in our population's selective environment (Rice et al., 2006)) of IV females that were exposed to males of different genetic backgrounds for either a “short” (3 hr) or a “long” (48 hr) period. By measuring the difference in fecundity between females in the two treatments for each of the male lines, we were able to estimate the relative negative impact (harm) that each male clone line had on female fitness (*see* Filice & Long, 2016). From these 26 lines, we chose the two lines with the greatest net effect on female fecundity (“high‐harm” males), and the two lines that had the lowest net effect on female fecundity (“low‐harm” males) for use in this study as we hypothesized that such lines would be most likely to influence the mate choice response of females, either due to the effect of the harm itself, or with other correlated phenotypic traits. Our use of the terms “high” and “low” harm is thus used to differentiate between males with different genetic backgrounds/phenotypes in our assays, and not to any one specific male trait.

### 2.2 Experimental protocol

We set out to examine the indirect genetic effects of mating experiences on female mate choice behaviors, and to quantify the amount of additive genetic variation underlying individual variation in female mate choice behaviors following different mating experiences. The experiment began by collecting female flies as virgins (within 8 hr of eclosion) from each of the 21 clone lines that were not used to generate either the low‐ or the high‐harm males. From each of these lines, we obtained 24 female hemiclones by crossing clone males to virgin IV females (as in Tennant et al., 2014). These flies were housed in groups of six (all containing members of the same hemiclone line) and were assigned to one of four “experience phase” treatments. Half the females derived from each hemiclone line were housed with males from a low‐harm hemiclonal background (low‐harm mating treatment) and half were housed with males from a high‐harm hemiclonal background (high‐harm mating treatment). Females were housed at a 2:1 male:female ratio (12 males and 6 females/vial). In half of the vials, females in each of the treatments described above remained together for 3 hr and were then kept separate from the males for 45 hr (short‐term exposure treatment) while the other half remained together for 48 hr until the start of the mate choice assay, allowing for multiple matings and continuous harassment to occur (long‐term exposure treatment). Thus, our “experience phase” treatments differ in terms of the amount and type of social and/or mating experience that females went though.

### 2.3 Mate choice assay

Following the “experience phase” of our experimental protocol, individual females were placed into mate choice chambers (using light CO_2_ anesthesia) in order to observe their subsequent mate choice behaviors (Supplementary Figure S1). These chambers consisted of a 41 × 41 × 8 mm main area (Fisher brand weighing boat 08‐732‐112) covered with a sheet of clear styrene (Evergreen Scale Models, Inc.), held in place with a bulldog clip, thereby creating an arena where females could freely move. Inside the arena, we installed four subchambers attached to the base of the main chamber (Micrewtube brand, Simport Scientific Inc.). One of these subchambers was filled with 60 μl of media for the female, and the other three caps were filled with 20 μl of media for males, who were physically blocked from the main chamber by a ½” (OD) 149‐micron polypropylene mesh disk (AmazonSupply.com). The mesh restricts the males from physically interacting with the female (and each other), but does not disrupt the exchange of olfactory and auditory (and possibly visual) signals between males and females (*see* Anderson, Kim, & Gowaty, 2007; Saltz, 2011). Thus, this arena design allows for females to sample (some) male display traits but eliminates the potential for male–male competition and harassment to confound the expression of a female's mate choice.

We placed males into their subchambers 18 hr before the start of the mate choice assay. In each mate choice chamber, we placed a single high‐harm male and a single low‐harm male (both from different hemiclonal backgrounds from the males used in the experience phase) into individual subchambers, and left the third subchamber empty (Supplementary Figure S1). All mate choice chambers were placed horizontally in a rack that permitted illumination from below (which ensured that females would appear in strong contrast to the background in our videos). We filmed chambers from above using JVC Everio GZ‐HM440U video cameras on a time‐lapse setting (1 frame s^−1^) for ~4 hr. Videos files were analyzed using the program VideoFly (Arbuthnott, Fedina, Pletcher, & Promislow, 2017) (generously provided by of Dr. Scott Pletcher, University of Michigan) which was used to track the physical position of the individual females in each frame of the video. Using this software, we counted the number of frames that each female spent on the surface of each subchamber containing either a “high‐harm male,” “low‐harm male,” or were elsewhere in the chamber (Supplementary Figure S1).

### 2.4 Statistical analysis

To understand female mate choice behaviors, we were interested in analyzing females’ overall interest in males and the expression of mate preferences. Interest in males was defined by the total amount of time (i.e., the number of frames) a female spent associating with males (both high‐ and low‐harm) compared to the entire duration of the assay. Preference was defined by the amount of time (number of frames) a female spent with high‐harm male compared to the amount of time spent with either male. Our decision to use “high‐harm” males as the preference numerator was arbitrary and the conclusions and implications of these results remain the same if we had instead used the “low‐harm” males at the numerator in our analyses.

All data analyses were conducted using R v3.1.2 (R Core Team, 2013). Data collected from hemiclonal females were analyzed using generalized linear mixed models (GLMMs), created using the *lme4* package (Bates, Mächler, Bolker, & Walker, 2014) with binomial response variables (examining the overall female attraction to males, and the female's preference for the harmful male) in both models. Models were fit and parameters were estimated using the Laplace approximation. Both models included mating treatment (high‐harm or low‐harm), male exposure length treatment (long‐term or short‐term), and their interaction as fixed effects, with hemiclone line (and all of it possible interactions with the previous effects) entered as random effects. Overdispersion in the response variables was accounted for by adding an observation‐level random effect, with a separate level for each individual measurement, as suggested by Browne, Subramanian, Jones, and Goldstein (2005).

The significance of fixed effects was first determined using log‐likelihood ratio (LLR) chi‐square tests implemented in the *Anova* function in the *car* package (Fox & Weisberg, 2011). Next, using the *bootMer* function, 95% CIs were calculated for each of the random‐effect variables based on 1,000 bootstrap samples. The statistical significance of each variance component was determined using a permutation test approach (Manly, 2007) whereby the magnitude of our model's variance component was compared to the distribution of 10,000 variance components each derived a randomized set of the experimental data.

In order to better understand the nature of the interaction between clone line and mating treatments in our model of female mate preferences, we calculated the mean proportion of time females from each clone line, in each of the two treatments (mated to high‐harm male or mated to low‐harm males) that was spent associating with the high‐harm male in the male choice chamber. We then calculated the correlation between these two variables, and obtained the standardized major axis (SMA) slope (Sokal & Rohlf, 2012) using the *lmodel2* package (Legendre, 2014). A SMA regression was calculated, as both the x‐ and y‐axes were subject to natural variation and measurement error. For each of these statistics, we calculated 95% CI by bootstrapping the data 1,000 times each using the boot function in the boot package (Canty & Ripley, 2016).

## 3 Results

**Figure 1:**
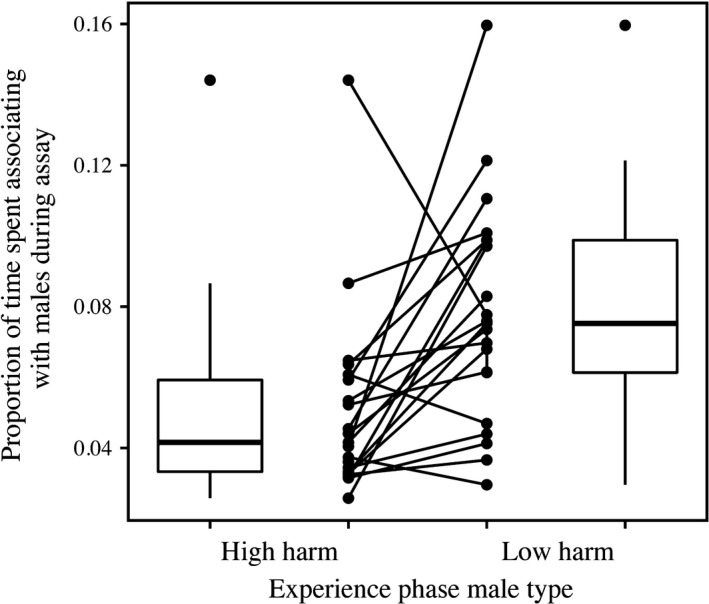
Effect of previous mating experience and female genotype on female interest in associating with males in *Drosophila melanogaster*. The reaction norm plot in the center depicts the proportion of time each female hemiclone line spent over a subchamber containing a male over the entire duration of the assay across the two mating experience treatments, while the boxplots depict the distribution of data independent of hemiclonal background. The boxes contain the middle 50% of data (interquartile range, IQR), and the horizontal lines represent the medians. Values >±1.5× IQR are outliers and are represented by closed circles, and all other values that are not outliers are represented by the whiskers above and below each box

When analyzing the degree of genetic variation associated with experience‐dependent mate choice behaviors, we found a significant effect of mating treatment on interest in males (LLR χ^2^ = 10.98, *df* = 1, *p* = .0009), with females that had been mated to “low‐harm” males showing greater levels of association with males than those females who had been mated to “high‐harm” males (Figure 1). We found no statistically significant effect of exposure length (LLR χ^2^ = 0.0974, *df* = 1, *p* = .7549) nor for the interaction between length and mating treatment (LLR χ^2^ = 0.1864, *df* = 1, *p* = .6659). When considering the random effects, we found that while clone line, the interaction between mating treatment and clone line, the interaction between length treatment and clone line, and the interaction between all three are statistically significant, they are of relatively small effect size with each only accounting for less than 2.5% of the observed phenotypic variation in the amount of time females spent associating with males (Table 1).

**Table 1:** Variance components estimated using a generalized linear mixed model (GLMM) fit by maximum likelihood (Laplace approximation) for hemiclonal *Drosophila melanogaster* female interest in associating with a male

Source of variance	Variance (*SD*)	Bootstrapped upper and lower 95% CI	% of variance explained	*p* value
Individual	1.049 (1.025)	1.199 0.901	46.89	.9999
Clone	0.050 (0.226)	0.163 0	2.27	.0047
Clone × male	0.043 (0.207)	0.136 0	1.92	.0194
Clone × length	0.041 (0.203)	0.140 0	1.84	.0276
Clone × male × length	0.054 (0.233)	0.149 0	2.41	.0207
Residual	1			

Females had previously been mated to either a “high‐harm” or a “low‐harm” male. The 95% CI values for the variance components were based on 1,000 bootstrapped samples of the data. The statistical significance of each variance component was determined using a permutation test approach (Manly, 2007) whereby the magnitude of each model's variance component was compared to the distribution of 10,000 variance components obtained from models by randomizing the identity of the original data.

**Figure 2:**
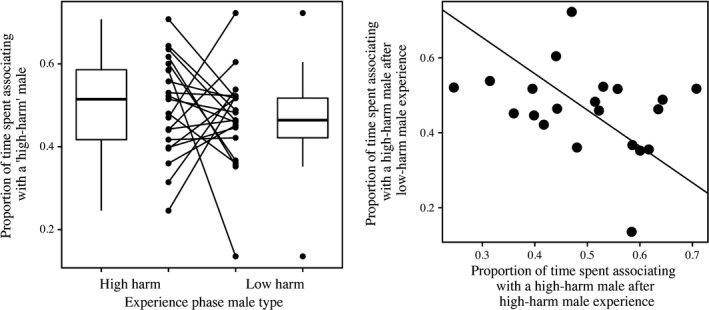
(a) *Left* effect of previous mating experience and female genotype on female preference in *Drosophila melanogaster*. The reaction norm plot in the center depicts the proportion of time each female hemiclone line spent with the high‐harm male over the total time she spent with males across the two mating experience treatments (degree of preference for high‐harm male), while the boxplots depict the distribution of data independent of hemiclonal background. The boxes contain the middle 50% of data (interquartile range, IQR), and the horizontal lines represent the medians. Values >±1.5× IQR are outliers and are represented by closed circles, and all other values that are not outliers are represented by the whiskers above and below each box. (b) *Right* scatterplot and regression line illustrating the negative relationship between the amount of time spent with the high‐harm male compared to females from each of 21 different hemiclone lines that had been previously exposed to either low‐harm males or high‐harm males

In our analysis of female preference data, neither of the fixed effects, nor their interaction was statistically significant (mating treatment: LLR χ^2^ = 1.8866, *df* = 1, *p* = .1696; length treatment: LLR χ^2^ = 0.2466, *df* = 1, *p* = .6195; interaction LLR χ^2^ = 0.6174, *df* = 1, *p* = .4320). Upon analysis of the random effects, clone line, the interaction between clone line and length treatment, and the three‐way interaction were not significant factors and explained none of the observed variation. However, the interaction between clone line and mating treatment was significant and explained ~9.72% of the observed phenotypic variation in female preferences (Figure 2a; Table 2). When we examined the behavior of the hemiclone females that had been mated to either high‐harm or low‐harm males, we found a significant, negative correlation between the two (PPMC correlation [bootstrapped 95% CI]: −0.300 [−0.022: −0.558]). Similarly, the SMA regression also has a significant negative slope (slope [bootstrapped 95% CI]: −0.968 [−0.444: −1.612]) (Figure 2b).

**Table 2:** Variance components estimated using a generalized linear mixed model (GLMM) fit by maximum likelihood (Laplace approximation) for hemiclonal *Drosophila melanogaster* female preference of harmful males

Source of variance	Variance (*SD*)	Bootstrapped upper and lower 95% CI	% of variance explained	*p* value
Individual	1.749 (1.322)	1.976 1.474	57.44	.9999
Clone	0 (0)	0.187 0	0.00	.2533
Clone × male	0.296 0.544	0.457 0.037	9.72	<.0001
Clone × length	0 (0)	0.079 0	0.00	.2293
Clone × male × length	0 (0)	0.144 0	0.00	.1895
Residual	1			

Females had previously been mated to either a high‐harm or a low‐harm male. The 95% CI values for the variance components were based on 1,000 bootstrapped samples of the data. The statistical significance of each variance component was determined using a permutation test approach (Manly, 2007) whereby the magnitude of each model's variance component was compared to the distribution of 10,000 variance components obtained from models by randomizing the identity of the original data.

## 4 Discussion

Individual variation in female mate choice can have important consequences for the direction and/or strength of the evolution of sexually selected traits by influencing the reproductive success of certain individuals (Jennions & Petrie, 1997). In many species, plasticity in the expression of mate choice behaviors may result from an individual's unique experiences (Verzijden et al., 2012; Rodríguez et al., 2013). Here, we demonstrate that behavioral plasticity in female mate choice behaviors is mediated by the IGEs associated with a previous mating experience. While previous studies have identified plasticity in mate choice behaviors as a result of social experience (e.g., Dukas, 2005; Rebar, Bailey, & Zuk, 2009; Rebar et al., 2011), we are the first (to the best of our knowledge) to uncover segregating genetic variation underlying this phenotypic variation. Our results indicate that the (1) indirect genetic effects associated with mating experience may shape subsequent female mate choice behaviors, (2) some of this variation is rooted in the presence of additive genetic variation in the population, and (3) the expression of this genetic variation is highly plastic (i.e., exhibits GxE effects). These findings provide novel insight into the causes and consequences of individual variation in female mate choice behaviors, and the potential of IGEs to interact with direct genetic effects to influence behavioral expression patterns.

### 4.1 Interest in males differs with identity of previous mate and interacts with genotype

When examining the effect of mating experience on subsequent female interest in males, we found that individuals that previously mated with “low‐harm” males subsequently spent almost twice the amount of time associating with chambers containing males compared to those females that previously mated with “high‐harm” males. We hypothesize that females in the “high‐harm” treatment were less interested in associating with males because they may have already incurred greater costs associated with male harassment and mating from their exposure treatment (Filice & Long, 2016; Holland & Rice, 1998), leading them to avoid further costs. This difference may also be associated with the toxic side effects of Acps which can reduce female longevity (Chapman et al., 1995; Rice, 1996) and receptivity to mating (Aigaki, Fleischmann, Chen, & Kubli, 1991; Chen et al., 1988). It is possible that the more harmful a previous mating experience was (i.e., increased exposure to the toxic side effects of Acps), the less receptive a female would be to the courtship of other males. If so, this experience‐dependent plasticity in female mate choice behavior may represent an adaptation that has evolved to maximize lifetime reproductive success, consistent with the model proposed by Fawcett & Bleay (2009). As the benefits associated with mate choice can frequently be context dependent (Qvarnström, 2001), a female that incurs lower direct physiological costs from a previous mate may benefit from remating if they are able to obtain higher “quality” sperm via sperm competition and/or cryptic mate choice (Dickinson, 1997; Jennions & Petrie, 2000). However, a female that incurs relatively higher direct physiological costs may not receive sufficient indirect benefits from remating to offset the costs (Gavrilets et al., 2001). Future studies should attempt to test whether this behavioral plasticity is an adaptation by comparing the lifetime reproductive fitness of females that have the opportunity to remate with their preferred male to females that are remated to the less‐favored male.

The presence of mate choice plasticity may also explain the maintenance of variation in deleterious alleles within a population's gene pool. In Filice and Long (2016), we showed that there is significant additive genetic variation associated with phenotypic variation in male‐induced harm. Our current study may explain how this variation is maintained: If females that have mated with a harmful male are less likely to remate, then one would predict increased frequency of alleles associated with male‐induced harm represented in the next generation. Johnstone and Keller (2000) suggested that the decreased female receptivity to males following remating is due to manipulation by the earlier mate, who may attempt to increase his share of paternity by reducing his partner's interest in other mates. Their model predicts that the size and potency of the manipulative substances transferred to females should increase in species with greater second‐male advantage, a phenomena well documented in *Drosophila* (Price, 1997; Price, Dyer, & Coyne, 1999). To test this hypothesis, future studies should quantify how female interest in males changes with time since mating, and its relationship to remating rates, female egg production, and the outcomes of sperm competition. Interestingly, we also observed a statistically significant interaction (albeit of small magnitude) between individual female genotype and mating treatment, which accounted for ~1.92% of the total observed phenotypic variation in interest in males (Table 2). This means that not all female lines which had mated to “high‐harm” males responded in the same manner. While most (18/21) of the hemiclonal lines spent more associating with males after having been exposed to “low‐harm” males than when exposed to “high‐harm” males, females from the remaining three lines exhibited the opposite plasticity. This heterogeneity further contributes to the observed individual variation in the population, and may help to maintain genetic variation in the population.

### 4.2 Female preferences vary with identity of previous mate and interaction with genotype

When looking at the effect of mating experience on female preferences (amount of time spent over the chamber containing one male phenotype compared to the other), we found no significant effects of either of our fixed effects (male type and exposure length treatments) or their interaction. However, we did find a significant interaction between female genotype and male experience which accounted for ~9.5% of all the observed phenotypic variation (Figure 2a). When this interaction was examined more closely, we found a negative correlation between the female preference phenotype exhibited by hemiclonal females that previously mated with a high‐harm male and females from the same hemiclonal line that had previously mated with a low‐harm male (Figure 2b). This means that those female genotypes that exhibited a strong preference for low‐harm males after having a previously mated with a “high‐harm” male also tended to exhibit a strong preference for high‐harm male they had been previously mated with a “low‐harm” male. This surprising result yields many exciting implications for our understanding of the causes and consequences of individual variation in female mate preferences. Firstly, it is consistent with the idea that plasticity in female choice behaviors reflects an interaction between the IGEs associated with a previous mate and the female's individual genotype. Secondly, it may also explain the maintenance of genetic variation in populations despite apparently strong directional sexual selection (i.e., the “lek paradox,” Kokko & Heubel, 2008). Theoretical models have predicted that GxE interactions between experience and genotype may act as a mechanism to maintain genetic variation (Ingleby et al., 2010; Kokko & Heubel, 2008), and our observations provide empirical support for such a mechanism. Travers et al. (2016) recently outlined the potential for female mating traits (mating and courtship latency) may change over a female's lifetime. Similar to our results, they found significant phenotypic variation in female mating latency between different families of fruit flies in both virgin and previously mated individuals. Our results would seem to indicate that some of this phenotypic variation is rooted in the interaction between individual genotype of the female and the IGEs of their previous mates. It is also worth considering the possibility that (some of) the variation we observed between the behavior of females from different hemiclone lines reflects additive genetic variation for their degree of preference for males perceived as phenotypically similar to their previous mates/social conspecifics (e.g., Zeh, Newcomer, & Zeh, 1998). While the “high‐” and “low‐”harm hemiclone males used in the “experience phase” of the assay were different from the two hemiclone lines used in the “mate choice” phase of the experiment, we cannot be sure that the phenotypic traits displayed by the males were (or were not) similar from the female's perspective. In *D. melanogaster*, virgin females appear to avoid mating with familiar males in favor of novel males (Ödeen & Moray, 2008), while in mated females, the opposite pattern has been observed (Tan et al., 2013). Our results suggest variation in this phenomenon may have a genetic basis, and is a potentially lucrative area of future research.

### 4.3 Behavioral plasticity as a form of resistance?

Our study's results have implications regarding the evolution of plasticity in mate choice behaviors and female resistance to male harm. Rodríguez et al. (2013) proposed five hypotheses that may explain the evolution of behavioral plasticity in mate preferences. All five of these hypotheses are explained by two general functions: first, that females alter their preferences to ensure mating and reduce the costs associated with mate choice (i.e., resource expenditure, time searching, predation risk); and secondly that females alter their preference to ensure mating with an “attractive” mate, or to prevent mating with an “unattractive” mate. While it is possible that our results support the latter function, we have no evidence to suggest whether males from the “high‐harm” line are more or less attractive than males from the “low‐harm” line (but see Friberg & Arnqvist, 2003). We have showed here that females could alter both their interest in prospective mates (receptivity) and preference based on the IGEs associated with a previous mate. Therefore, we suggest that plasticity in mate choice behaviors may potentially operate as a means of female “resistance” to male‐induced harm. In previous studies demonstrating the presence of genetic variation in female resistance (Linder & Rice, 2005; Lew et al., 2006) and its adaptive basis (Holland & Rice, 1999; Wigby, & Chapman, 2005), the actual mechanisms that mediate female resistance were not specifically characterized. Holland and Rice (1998) suggested in their “chase‐away” sexual selection hypothesis that females may resist the direct costs of mating by evolving biases against traits that stimulate them to mate. Since then, theoretical models have inferred that females might evolve specific mate choice behaviors as a means of reducing the direct costs of mating (Gavrilets et al., 2001) and that female plasticity can reduce the manifestation of sexual conflict (McLeod & Day, 2017), but to date few studies have attempted to empirically test these hypotheses (Moore, Gowaty, Wallin, & Moore, 2001; Moore et al., 2003). Therefore, it is integral for future studies to continue investigating the dynamics between sexual conflict theory and mate choice. In order to better understand the evolutionary significance of our results, studies should consider how individual components of harm (i.e., Acp concentrations, physical condition) might influence subsequent mate choice, and how female fitness is affected by changes in their mate choice behaviors.

## 5 Conclusions

In this experiment, we identified the IGEs associated with mating experience as a novel source of variation in female mate choice behaviors in the model species *D. melongaster*. We also identified an interaction between mating experience and individual genotype (a GxE effect) on interest in males and female preferences, suggesting that a female's preference and interest in males is highly plastic. Our results offer new insight into the maintenance of variation in male traits and the evolution of plasticity in female mate choice behaviors.

## Conflict of Interest

None.

## Data Accessibility

The data and codes have been deposited to Dyrad: https://doi.org/10.5061/dryad.6008n.
