# Supplementary figures and images for: Phenotypic plasticity in female mate choice behavior is mediated by an interaction of direct and indirect genetic effects in Drosophila melanogaster
Source: Ecol Evol. 2017 Apr 9;7(10):3542–51. doi: 10.1002/ece3.2954 (PMC5433979; doi:10.1002/ece3.2954)

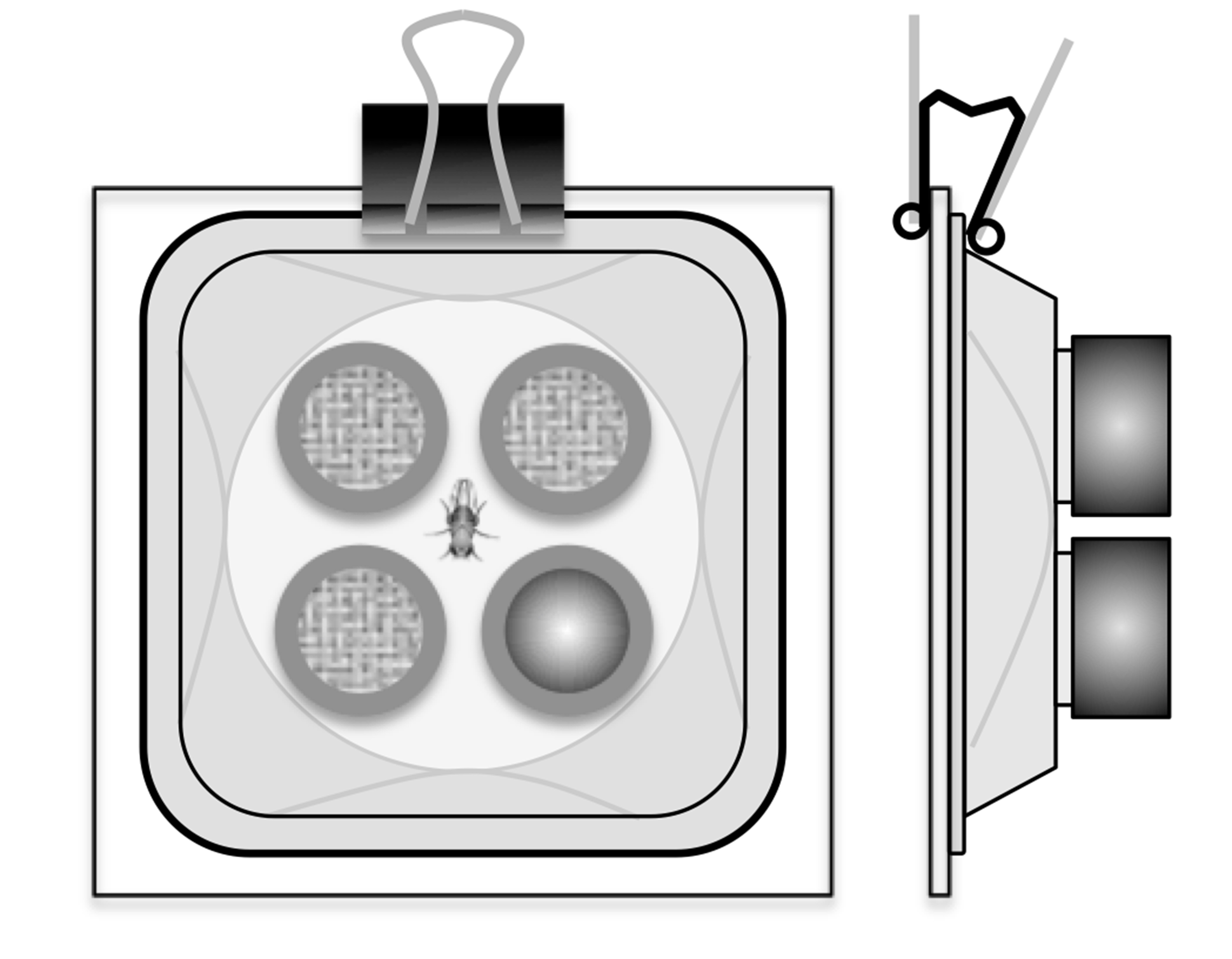

Supplement: Supplementary file 1 [file ECE3-7-3542-s001.jpg]
